# Manipulation of artificial light environment improves plant biomass and fruit nutritional quality in tomato

**DOI:** 10.1016/j.jare.2024.11.030

**Published:** 2024-11-24

**Authors:** Ying Zhang, Kangyou Zhu, Xiujie Wang, Jiarong Yan, Haiyan Zhu, Nan Zhang, Yiting Wang, Qi Zhao, Yanan Liu, Xin Bu, Chenghao Jiang, Xin Sun, Golam Jalal Ahammed, Shuyu Cai, Sida Meng, Zhouping Sun, Mingfang Qi, Tianlai Li, Feng Wang

**Affiliations:** a College of Horticulture, Shenyang Agricultural University, Shenyang 110866, China; b Key Laboratory of Protected Horticulture, Ministry of Education, Shenyang 110866, China; c College of Land and Environment, Shenyang Agricultural University, Shenyang 110866, China; d College of Horticulture and Plant Protection, Henan University of Science and Technology, Luoyang 471023, China; e School of Architectural Engineering, Shaoxing University Yuanpei College, Shaoxing 312000, China

**Keywords:** Red light, Blue light, Photosynthesis, Fruit quality, Photoperiod, *Solanum lycopersicum* L

## Abstract

**Introduction:**

The yield and quality of tomato (*Solanum lycopersicum.* L) are often decreased when plants suffer from low light intensity and short-photoperiod in winter. Manipulation of the artificial light environment is a feasible technology to promote off-seasonal production and improve fruit nutritional quality in the greenhouse.

**Objectives:**

Here we aim to investigate the appropriate balance between red (R) and blue (B) light to improve tomato yield and quality traits.

**Methods:**

Biochemical, molecular and genetic analysis were used to study the photosynthetic traits, pigments, sugar and volatile accumulation pathway genes.

**Results:**

Tomato plants subjected to R1B0.8, a ratio between monochromatic red (R) and blue (B) light, for 16 h photoperiod showed significantly increased chlorophyll and biomass accumulation compared to white (W) light treatment. R1B0.8 light treatment enhanced electron transfer efficiency and photosynthetic capacity by improving the light energy utilization rate and inducing photosystem core subunit genes (*SlPsaC, SlPsaB, SlPsaA*) and light-harvesting complex genes (*SlLHCB/A*). Compared to W light, R1B0.8 light also induced carotenoid accumulation and accelerated fruit ripening, which was associated with the upregulation of carotenoid biosynthesis genes (*SlPSY1, SlPDS*) and ethylene biosynthesis genes (*SlACS2, SlACO2*) in tomato fruits. Moreover, fruits exposed to R1B0.8 light treatments significantly improved fructose and glucose accumulation and the expression of the volatile-related gene (*SlAADC1a)* and flavor-related gene (*SlGORKY*).

**Conclusion:**

Our results showed that R1B0.8 light with a 16 h photoperiod could prominently promote photosynthetic traits, pigments, sugar and volatile accumulation in tomato. Our findings on the manipulation of artificial light environments in protected horticulture offer possibilities for enhancing crop yield and quality to meet the increasing global demand for food.

## Highlights

• Red and blue light increases photosynthesis and plant biomass through a *LHCB/A* and *PsaC/B/A* modules in tomato*.*
• Manipulation of light quality and photoperiod enhances carotenoid accumulation and fruit ripening in tomato.
• Suitable light quality and photoperiod induces carotenoid (*PSY1, PDS0*) and ethylene (*ACS2, ACO2*) related-genes’ expression.
• R1B0.8 light promotes fructose and glucose accumulation and the metabolism of volatiles in a *SlAADC1a*-dependent manner.

## Graphical abstract

**Figure:**
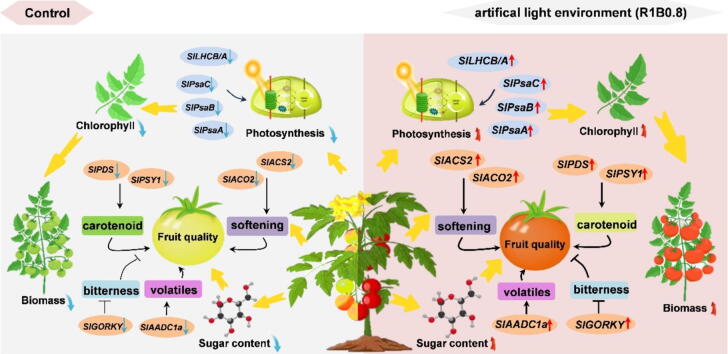

## Introduction

Light as a signal and energy source plays an essential role in plant development and physiological responses, providing needed energy for photosynthesis and useful information for regulating plant metabolism [1]. Plants frequently monitor changes in the dynamic fluctuations in light environments and fine-tune their growth patterns and development correspondingly to optimize fitness. Plants have evolved a sophisticated array of photoreceptors in response to the ever-changing environment [2], [3]. Red (R, 600–700 nm) and far-red (FR, 700–750 nm) light spectra are sensed and perceived by phytochromes (phyA-phyE). Phytochromes possess two forms of mutual conversion, Pr and Pfr, absorbing the R and FR light, respectively [4]. Blue light (B, 315–400 nm) is detected by cryptochromes (CRY1, CRY2 and CRY3), phototropins (PHOT1, PHOT2) and F-box containing flavin-binding proteins (ZEITLUPE, FKF1/LKP2) [5], and UV-B light (280–315 nm) is absorbed by UV RESISTANCE LOCUS 8 (UVR8) receptor [6].

Different morphological and physiological responses are influenced by specific spectra. R and B are the two most effective spectral wavelengths for plant growth because of their pivotal function in plant photosynthesis [7]. B light is reported to inhibit stem elongation, induce stomatal opening and photosynthetic pigment accumulation, and decrease leaf area [8]. In addition, increasing the percentage of B light within a certain range during plant growth and development can improve the photosynthetic ability of a range of plant species including lettuce [9], cucumber [10] and rapeseed [11]. Notably, B light significantly enhances the accumulation of plant secondary metabolites, such as total phenolic, anthocyanins, and flavonoids [12]. Moreover, B light can promote ascorbate synthesis by deactivating the PAS/LOV photoreceptor, which inhibits GDP-L-galactose phosphorylase [13]. B light-induced accumulation of anthocyanin is regulated by a FaCRY1-FaCOP1-FaHY5 transduction pathway in strawberry [14]. R light influences the chlorophyll content and photosynthetic apparatus, regulates cell expansion and growth, and promotes hypocotyl elongation through phytochromes in plants [15]. Overall, manipulating the light spectra could trigger potential benefits by promoting plant growth, development and specialized metabolite synthesis.

Previous reports suggested that plants exposed to monochromatic R or B light during long periods could be insufficient to maintain the requirements of vegetative growth and reproductive development. Plants show abnormal leaf morphology and decreased photosynthetic capacity and biomass when R light is supplemented alone. Therefore, B light is added in the presence of R light to relieve these symptoms in plants [16]. Hence, manipulating the R/B ratio is an important manner to enhance the growth and development of plants. Compared to R alone, a ratio of R and B light is necessary to increase biomass accumulation and leaf numbers in tomato plants [17]. R/B light (R: B = 7:2) can improve the photosynthetic rates, promote sucrose, fructose, glucose, and starch accumulation, and enhance chlorophyll and carotenoid accumulation in cucumber [18]. Under the combination of R, B and W (R1W1B0.5) light treatments, photosynthesis, biomass and fruit quality are significantly improved in tomato via photoreceptors (PHYB1 and CRY1)-SlHY5-SlLHCA/B/SlCYCB pathway [19]. Nonetheless, the most appropriate proportion of R and B light required for better growth and development of tomato remains to be further explored.

When fruits are exposed to a low light environment, they frequently exhibit some abnormal phenomena such as color disorder and deterioration of fruit quality [20]. However, there is still a lack of knowledge on how light quality affects fruit quality. The transition to fruit ripening is associated with the conversion of chloroplasts into chromoplasts leading to chlorophyll degradation and carotenoid (β-carotene and lycopene) accumulation [21]. Carotenoid, a diverse group of natural pigments characterized by their yellow, orange, and reddish hues, play a crucial role in photosynthesis and act as attractants for pollination in plants [21], [84]. Additionally, these pigments are important for imparting the bright colors observed in ripened tomato fruits. Previous reports have illustrated that R activates PHY to inhibit the accumulation of PHYOCHROME INTERACTING FACTOR 1a (PIF1a) proteins that bind to the phytoene synthase 1 (*PSY1*) promoter to stimulate the expression of *PSY1* and induce the accumulation of lycopene [22]. B light is also reported to be involved in increasing lycopene accumulation in tomato fruits by inducing the transcript levels of the *CRY1a* gene [23]. In addition, light quality combinations have generally been observed to participate in improving the fruit quality in tomato fruits. For instance, the combination light (R: B = 3:1) can improve the accumulation of soluble solids, and soluble sugars through increasing the abundance of proteins associated with glucose metabolism pathways in tomato fruits [24]. Similarly, R and B mixed lights are important for improving lycopene biosynthesis and carbohydrates in tomato fruits [25]. However, the mechanisms of manipulation of the different light spectral compositions for improving fruit ripening and quality in tomato are not well understood.

Recent advances in artificial light technology, especially LED, enable crops’ lighting regimes to be precisely manipulated in greenhouses. Compared to traditional light sources, LED light has many advantages, such as low energy consumption, high light efficiency, high security and controlled light wavelengths [9]. Manipulation of the artificial light environment with LED lights could increase the density of plant cultivation, shorten the plant growth time, and significantly improve the space utilization efficiency of plant cultivation. The changes in light conditions (including photoperiod and light quality) have a profound impact on the morphogenesis and physiological metabolism in plants [26]. Thus, we hypothesize that an appropriate R/B ratio and photoperiod will significantly improve the yield and quality of tomato. Different light quality and photoperiod treatments were used to confirm this. Here, we found that a combination of R and B light (R1B0.8) promoted photosynthetic pigment accumulation, enhanced the photosynthesis, light capture ability of the photosystem, and electron transfer efficiency via inducing the expression of photosystem core subunit genes (*SlPsaC, SlPsaB, SlPsaA*) and *LIGHT-HARVESTING COMPLEX B* and A (*LHCB/LHCA*), thereby increasing the accumulation of biomass in tomato. Moreover, R1B0.8 treatments up-regulated the expression of ripening-related genes resulting in accelerated color transition and ripening of tomato fruits. Subsequently, R1B0.8 light promoted the soluble sugars accumulation and the expression level of the volatile-related gene *SlAADC1a* and flavor-related gene *SlGORKY*. Our results also showed that the photoperiod (16/8h, light/dark) is suitable for tomato growth and development. Collectively, the study illustrated that a combination of R and B light plays an essential role in photosynthesis and fruit quality in tomato, which provides a new insight into the mechanism of enhancing plant growth and development in tomato.

## Materials and methods

### Plant materials and growing conditions

Tomato (*Solanum lycopersicum* L.cv. Ailsa Craig and Liaoyuanduoli) seedlings were grown in pots with a mixture of peat and vermiculite in a ratio of 3:1 (v/v), receiving Hoagland nutrient solution [27]. Soil moisture sensors (MAS-1, Decagon Devices, Pullman, USA) were used to monitor pot volumetric water content (VWC) and to regulate an irrigation system to maintain 40–50 % VWC. Seedlings were cultivated at 25 °C/20 °C (day/night, 12 h photoperiod) in a growth chamber (Ningbo Jiangnan Instrument Factory, Ningbo, China) at 200 μmol m^-2^s^−1^ light intensity with 60 % humidity until the five-leaf stage.

### Light treatments

**Fig. 1:**
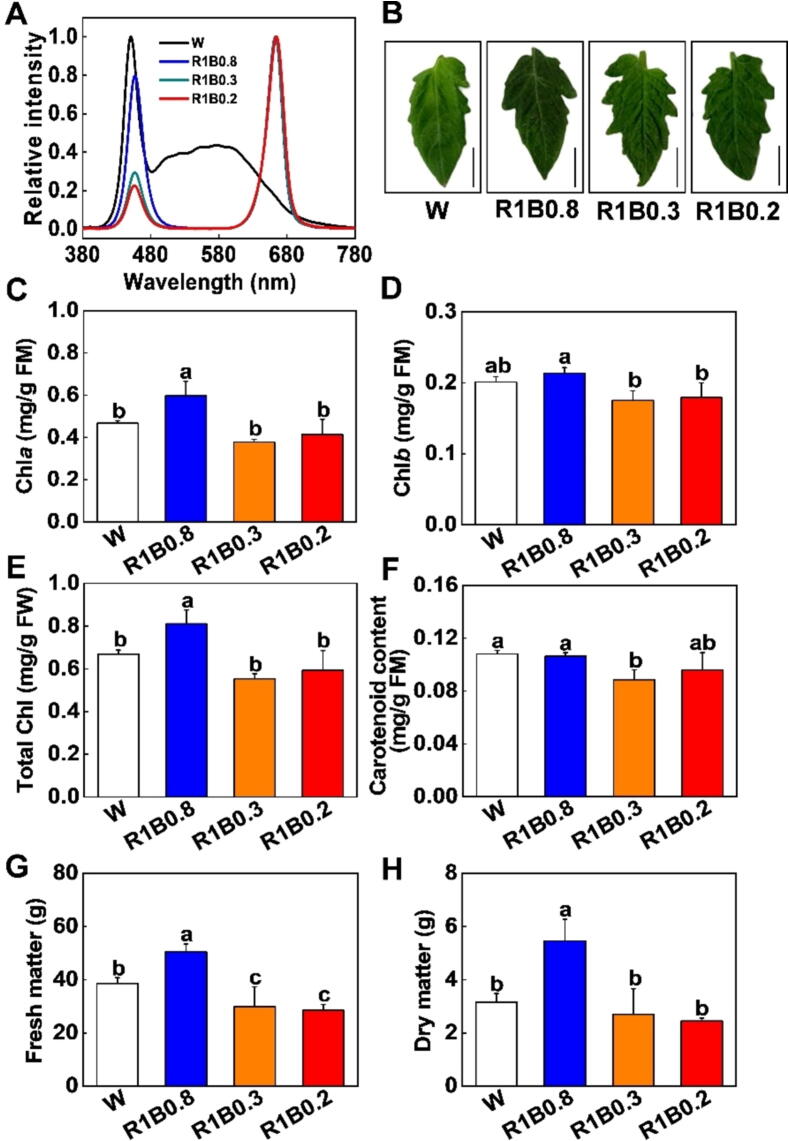
**Red (R) and blue (B) mixed light affected photosynthetic pigment contents and biomass in tomato (Alisa Craig) plants**. (A) Spectral composition of different light treatments. (B) Phenotypes of tomato leaves grown under white (W, 400–700 nm), red-blue (R1B0.8, R1B0.3 and R1B0.2) light treatments. Bars in (B), 2 cm. (C-F) chlorophyll *a* (Chl *a*; C), chlorophyll *b* (Chl *b*; D), total chlorophyll (Total Chl; E), carotenoid contents (F) in tomato leaves after exposure to W, R1B0.8, R1B0.3 and R1B0.2 light treatments for 12 d. (G-H) After 12 d of light treatment fresh matter (FM; G) were measured. The dry matter (DM; H) were measured after the light treatment samples were heated at 105 °C for 30 min and then dried to constant weight at 80 °C for 4 days. Values are expressed as the mean ± SD (n = 3). n = 3 independent biological replicates. At least six seedings leaves or fruits were harvested for each biological replicate. Statistical significance is annotated by different letters (*p* < 0.05).

Different light treatment experiments were performed in controlled environment growth chambers with the same intensity at 200 µmol m^−2^ s^−1^. The distance between the LED (FuJian Sananbio Technology Co., Ltd, China) light source and seedlings was 15 cm. There were four layers in the growth chamber with the same temperature, relative humidity and air velocity, but the light quality was different in every layer with different LED light. Seedlings or fruits were placed in three separate compartments on each layer. The light quality treatments were as follows: red (R): blue (B) light at 1:0.8 (R1B0.8), 1:0.3 (R1B0.3), 1:0.2 (R1B0.2), and white (W) light as a control (Fig. 1A). The combinations of B (λ_max_ = 445 nm) and R (λ_max_ = 665 nm) lights spectra ratios were measured by Lighting Passport (Asensetek Inc., China). Five-leaf stage seedings were placed in different light conditions (W, R1B0.8, R1B0.3, R1B0.2) for 12 days. In addition, five-leaf stage seedings were also grown at R1B0.8 light quality conditions with four different photoperiods (8/16 h, 12/12 h, 16/8h, and 24/0h, day/night) treatments for 12 days.

For the fruit experiment, flowers were marked on the day of anthesis to determine fruit developmental and ripening stages [28]. When tomato fruits reached the mature green stage (MG, full-size green fruit, approximately 35 days after anthesis, and fruit dimensions not expansion) in greenhouse [29], fruits of the same size and ripeness were harvested and transferred to a growth chamber with different light quality (W, R1B0.8, R1B0.3, and R1B0.2) treatments to do postharvest experiments for 10 days at 25 °C /20 °C (day/night, 12 h photoperiod) conditions with 60 % humidity. After treatment, seeds, jelly, and placenta were discarded from each fruit and the pericarp was sampled from around the equator of the fruits and then quickly stored in a −80 °C freezer.

The experimental design was a completely randomized design with three replicates. Leaves and fruits were harvested from at least six seedlings or plants per biological replicate. All experiments were repeated three times independently with similar results. All samples were collected from the seedlings in the middle area of the tray to minimize the effects of light reflection from the walls.

### Measurements of pigment contents

The leaf photosynthetic pigment (chlorophyll, carotenoid) contents were measured as described by the previous report [19], fresh leaves (0.3 g) under all treatments were added into the tube with 10 mL anhydrous ethanol: 10 mL 80 % acetone (1:1, v/v), and incubated under darkness environment for 24 h until samples fade to white. The data was recorded at the absorbance of 663 nm, 645 nm, and 470 nm with a spectrophotometer (Shimadzu, Japan). The pigment was calculated according to the previous report [30].

For ProtoIX, MgProtoIX, and Pchlide analysis, we randomly selected 0.5 g leaves (fresh matter, FM) and homogenized them into an ice-cold mortar using a 25 mL of 8:2 (v/v) mixture of acetone and 0.1 mol L^−1^ NH_4_OH for 24 h under dark conditions. Next, the homogenate was 4 °C centrifuged at 10087 rpm for 10 min and the supernatant was collected into a new tube. The absorbance of the supernatant at 450 nm, 590 nm, and 575 nm was determined immediately with a spectrophotometer. The concentrations of ProtoIX, MgProtoIX, and Pchlide were quantified according to the previous report [19].

Lycopene was extracted from 0.5 g fresh fruit pericarps by adding 5 mL hexane: ethanol: acetone (2:1:1, v/v/v), with 0.05 % butylated hydroxytoluene previously added in acetone, followed by shaking for 15 min. Then 1.5 mL water was added and shaken for 2 min. Afterward, the samples were centrifuged at 10087 rpm for 1 min to obtain the upper layer solution (hexane). At last, the supernatant was determined by monitoring the absorbance at 503 nm using a spectrophotometer. The lycopene content of each sample was calculated as described by the previous report [31].

### Measurements of fresh and dry matter

After 12 d of light treatment, tomato seedlings were completely taken out from pots together with the soil and root system, the shoot and root were cleaned with water then blotted dry and weighed separately to determine their fresh matter (FM). All samples were heated at 105 °C for 30 min, and then dried to constant weight at 80 °C for 4 days before measuring their dry matter (DM). A semi-analytical electronic balance (Sartorius, Germany) was used to weigh these samples.

### Measurements of Gas Exchange, and Chlorophyll Fluorescence

The net photosynthetic rate (Pn), transpiration rate (Tr), intercellular CO_2_ concentration (Ci) and stomatal conductance (Gs) were determined on the fourth fully expanded mature leaves (counting from the bottom of the plant; the first true leaf was defined as leaf 1) during 9:00 to 11:00 with an LI-6400 (LI-COR, Inc., Lincoln, NE, USA) as described [27], [32], [33]. The measured conditions were a CO_2_ concentration of 400 μmol mol^−1^, and a light intensity was 631 μmol m^−2^ s^−1^[2], [27]. Leaves from at least six different seedlings were measured per biological replicate. All experiments were repeated three times independently with similar results.

Chlorophyll *a* fluorescence was obtained by DUAL-PAM-100 (Heinz Walz, Effeltrich, Germany). To monitor the rapid light curves (RLCs), we used actinic light (AL, 635 nm) of 10, 18, 36, 94, 172, 214, 330, 501, 759, 1178, 1455 μmol photons m^−2^ s^−1^. The duration of each light intensity was 30 s and the saturation pulse was 10000 μmol m^−2^ s^−1^ for 300 ms. After plants were dark-adapted for 30 min, the following chlorophyll fluorescence parameters were determined: electron transport rates (ETR), effective quantum yield of PSII [Y(II)] and PSI [Y(I)], photochemical quenching (qP) and maximum quantum yield of PSII (Fv/Fm) [27], [34], [35].

For the OJIP curve, the leaves were irradiated with high-intensity actinic light (10000 μmol photons m^−2^ s^−1^), and the chlorophyll fluorescence showed an instantaneous increase. The fluorescence transient showed a polyphasic rise including phases O, J, I, and P, and the four characteristic points correspond to the fluorescence values at 0 ms, 2 ms, 30 ms and 1000 ms. This curve provides more information about electron transfer on the donor side and acceptor side of PSII [36], [37]. The original measured data was used for calculation according to the JIP-test previously described equations [27]. The analyzed parameters were described in Supplementary Table S2.

### Determination of fruit color and firmness

When tomato fruits reached the mature green stage (MG, full-size green fruit, approximately 35 days after anthesis, and fruit dimensions not expansion) in greenhouse [29], fruits of the same size and ripeness were harvested and transferred to a growth chamber with different light quality (W, R1B0.8, R1B0.3, and R1B0.2) treatments to do postharvest experiments for 10 days at 25 °C /20 °C (day/night, 12 h photoperiod) conditions with 60 % humidity. After different light treatments for 10 days, the fruit color changed. Then the color values of tomato fruits were measured using the D65 illuminant with a colorimeter (Konica Minolta CR-400). The color index was calculated according to the a^∗^/b^∗^ ratio [19].

The assessment of fruit firmness was assessed with a fruit texture testing machine (CT3, Brookfield Inc., Middleboro, USA), The equatorial part of the fruits was inserted at a final depth of ∼7 mm by a puncture test with a 2-mm cylindrical probe, and the force measurements were recorded. The measurement was repeated on at least six fruits.

### Determination of soluble sugar

Fresh fruit pericarps samples (1 g) were mixed with 5 mL of 80 % ethanol by sonication for 60 min at 80 °C. The extracted sample was subsequently centrifuged at 9208 rpm for 5 min and the supernatant was transferred to a new 15 mL tube. After extraction, 5 mL of the supernatant was evaporated in an 80 °C water bath until complete dryness. The residue was redissolved in 1 mL of distilled water and then filtered through a 0.45 μm nylon organic filtration. Sucrose, glucose and fructose were measured using high-performance liquid chromatography (HPLC) (Waters 600E) with a Sugar-D column (250 × 4.6 mm) at a flow rate of 1.0 mL min^−1^. The soluble sugar was identified by their retention times and was quantified according to the peak area. Soluble solids content in tomato juice from all light treatments was determined by using a hand-held refractometer (Master Series, 0–30 %, Atago) [38].

### qRT–PCR analysis

The total RNA was isolated from tomato leaves and fruits using the Plant Total RNA Extraction Kit (Tiangen, Beijing, China) according to the manufacturer's instructions. The first-strand complementary DNA (cDNA) was reverse transcribed from 1 µg of total RNA using a HiScript II reverse transcriptase kit (Vazyme, Nanjing, China). The expression levels of target genes were detected using 2 × Universal SYBR Green Fast qPCR Mix (ABclonal, Wuhan, China), analysis using the CFX96TM Real-Time PCR System (Bio-Rad), three replicates per treatment, *ACTIN2* gene was used for internal control. Primers used in the study for PCR are listed in Supplementary Table S1 [19], [39].

### Statistical analysis

All statistical analyses were performed using SPSS 22.0 and Microsoft EXCEL 2019. Data are expressed as means ± SD. Significant differences among all treatments were analyzed by ANOVA followed by Tukey’s test at *p* < 0.05. The figures in our study were drawn with Origin 2023.

## Results

### Artificial light sources promote photosynthetic pigments, fresh and dry matter accumulation in tomato

Different varieties of tomato plants, such as Alisa Craig (AC) and Liaoyuanduoli (LYDL) were incubated under different light quality [W, R: B = 1:0.8 (R1B0.8), R: B = 1:0.3 (R1B0.3), R: B = 1:0.2 (R1B0.2)] (Fig. 1A). Unaided visual observation revealed that the leaves of tomato plants grown under R1B0.8 light exhibited the greenest phenotype compared with other treatments (Fig. 1B). Subsequently, photosynthetic pigment, such as the total chlorophyll, chlorophyll *a* and chlorophyll *b* contents were measured in the leaves under R1B0.8 treatments, which were remarkably higher than those under other treatments. However, no significant differences in total chlorophyll were observed among W, R1B0.3, and R1B0.2 treatments. These results demonstrated that an appropriate increase in B light ratio under consistent R light conditions could enhance the chlorophyll accumulation in both AC and LYDL tomato plants (Fig. 1C-E; Supplementary Fig. S2A-C). However, in plants grown under R1B0.8 treatments, the carotenoid content in leaves was not significantly different from W light (Fig. 1F; Supplementary Fig. S2D). Compared with the tomato seedlings grown under W, R1B0.3 and R1B0.2 treatments, the total fresh matter (FM) and dry matter (DM) of AC and LYDL tomato plants were significantly increased by R1B0.8 light (Fig. 1G, H; Supplementary Fig. S1A-D; Supplementary Fig. S2E-F). Tomato leaves grown under R1B0.8 light had the highest contents of Pchlide, Mg-ProtoIX, and ProtoIX compared to other light treatments (Supplementary Fig. S3A-C). The above results indicate that increasing B light appropriately under R light conditions can promote chlorophyll accumulation and fresh and dry matter production in tomato seedlings.

### R and B mixed lights influence the chlorophyll fluorescence in tomato

**Fig. 2:**
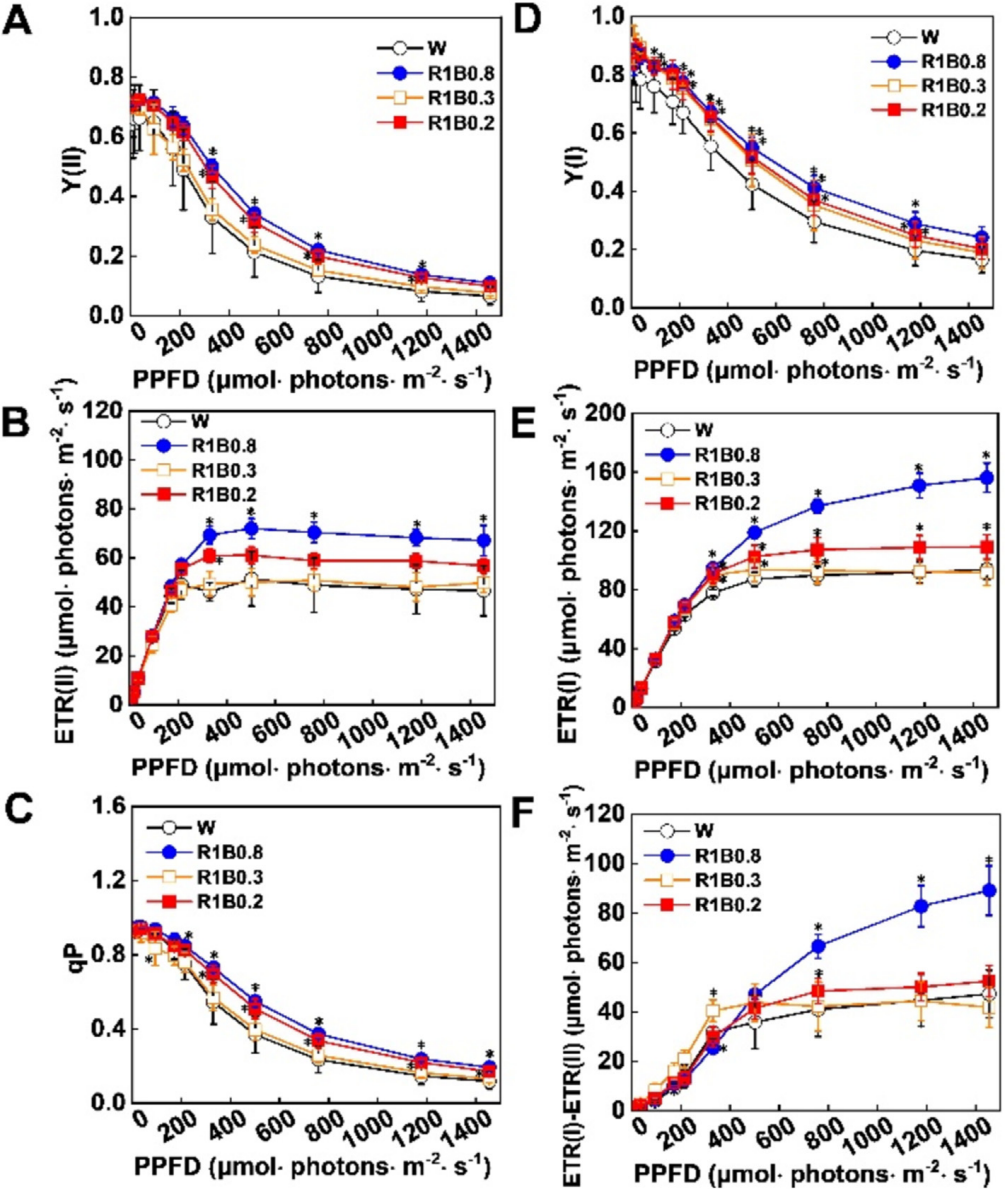
**Various light environments regulate electron transport rates in tomato seedlings**. (A-C) Changes in PSII parameters, including the effective quantum yield of PSII [Y(II); A], the electron transport rates of PSII [ETR(II); B], and the photochemical quenching coefficient (qP; C) after tomato leaves exposed to W, R1B0.8, R1B0.3 and R1B0.2 light treatments for 12 d. (D, E) Changes in PSI parameters, including the effective quantum yield of PSI [Y(I); D] and the electron transport rates of PSI [ETR(I); E] in tomato leaves under different light treatments for 12 d. (F) Changes in the cyclic electron flow around PSI [ETR(I)–ETR(II)] in tomato leaves were measured on 12 d under W, R1B0.8, R1B0.3, and R1B0.2 treatments. Values are expressed as the mean ± SD (n = 3). n = 3 independent biological replicates. At least six seedings leaves or fruits were harvested for each biological replicate. Asterisks indicate significant difference (*p* < 0.05).

Given the difference in photosynthetic pigment in different light quality, we speculated that light quality treatments might have an important impact on photosynthetic parameters in tomato seedlings. Thus, we analyzed the change in fluorescence parameters to evaluate the impacts of different R/B ratios on the PSI/PSII activity in tomato leaves. The Y(I) and Y(II) (The effective quantum yield of photosystem) under R1B0.8 treatments were significantly greater than other W, R1B0.3 and R1B0.2 treatments (Fig. 2A, D). Furthermore, electron transfer rates of both PSII and PSI [ETR(II), ETR(I), respectively] reached the highest value in tomato seedlings subjected to R1B0.8 light (Fig. 2B, E). [ETR(I)-ETR(II)] showed a similar trend with ETR(I) and ETR(II) (Fig. 2F). Consistently, photochemical quenching (qP) was increased by both R1B0.8, R1B0.3, R1B0.2 treatments compared with W light, especially under R1B0.8 treatments (Fig. 2C). These data show that R1B0.8 treatments might have resulted in higher maximum photochemical efficiency and electron transport capacity, which is consistent with increased photosynthetic pigment content.

**Fig. 3:**
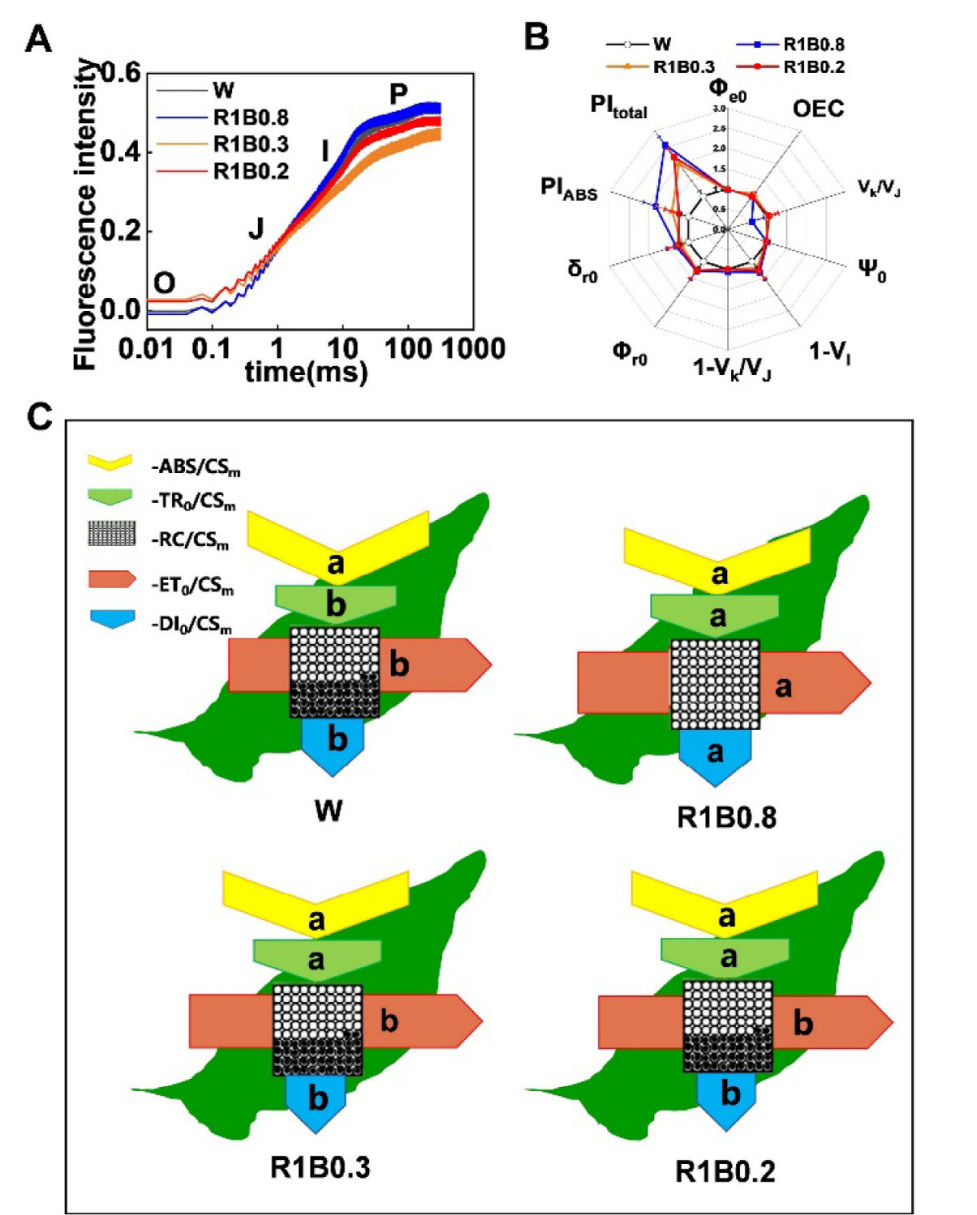
**R and B mixed light affects the photosystem II activity of tomato**. (A) OJIP curves. (B) Radar plots of JIP parameters calculated from OJIP transient curves of tomato leaves under W, R1B0.8, R1B0.3, R1B0.2 light treatments. (C) Energy pipeline model of phenomenological fluxes per cross-section as affected by W, R1B0.8, R1B0.3, and R1B0.2 light treatment. The area of different colored arrows, such as yellow, green, red and blue arrows, represented the high or low of the values of ABS/CS_m_, TR_0_/CS_m_, ET_0_/CS_m_, DI_0_/CS_m_, respectively. Values are expressed as the mean ± SD (n = 3). n = 3 independent biological replicates. At least six seedings leaves or fruits were harvested for each biological replicate. Statistical significance is annotated by different letters (*p* < 0.05).

Chlorophyll *a* fluorescence transient (OJIP transient) can provide information about energy absorption, distribution and utilization in photosynthesis, as well as information on primary photochemical reactions in PSII reaction centers [40]. Our data found that the fluorescence intensity had a slight decrease under R1B0.8 treatments compared to W, R1B0.3 and R1B0.2 before the J phase (Fig. 3A). In contrast, plants grown under R1B0.8 treatments had a higher fluorescence intensity between steps J to P (Fig. 3A), which implied that R1B0.8 treatments could promote the electron transfer from Q_A_ to Q_B_ of PSII. Next, we analyzed the different light quality-induced changes in biophysical parameters by JIP-test (Fig. 3B). In particular, we found that R1B0.8 treatments induced a significant increase in the photosynthetic performance for energy conservation from PS II absorption photons to the reduction of intersystem electron acceptors (PI_ABS_) and performance up to the reduction of PSI terminal electron acceptors (PI_total_), but unaffected on oxygen-evolving complex (OEC) activity (Fig. 3B; Supplementary Table S2), indicated that R1B0.8 treatments promoted D1 protein turnover and enhanced Q_A_ fixation and stability of PSII, and then enhanced photosynthetic electron transport of PSII and PSI.

In order to elucidate the various light quality-induced changes in PSII efficiency, energy pipeline leaf models under W, R1B0.8, R1B0.3, and R1B0.2 light treatments were constructed. The area of different colored arrows, such as yellow, green, red and blue arrows, represented the high or low values of absorption flux (ABS/CS_m_), trapped energy flux (TR_0_/CS_m_), electron transfer (ET_0_/CS_m_), dissipated energy flux (DI_0_/CS_m_), respectively. As shown in Fig. 3C, compared with W treatments, the area of the blue patch, which represented the value of dissipated energy flux (DI_0_/CS_m_), was larger under R1B0.8 treatments. Furthermore, plants grown under R1B0.8 treatments remarkably increased the TR_0_/CS_m_ and the electron transfer ET_0_/CS_m_ in tomato leaves (Fig. 3C). However, ABS/CS_m_ had no significant change under all treatments (Fig. 3C). Empty and full black circles represented that the percentage of active (Q_A_ reducing) and non-active (non-Q_A_ reducing) reaction centers of PSII, respectively. Strikingly, the number of empty circles reached the highest value under R1B0.8 treatments, indicating R1B0.8 light induced a remarkable increase in RC/CS_m_ (the density of active reaction centers) (Fig. 3C). These results suggest that R1B0.8 treatments can significantly promote PSII performance by enhancing the efficiency of PSII energy capture and increasing the activity of PSII reaction centers.

### Light quality manipulation improves photosynthesis in tomato

**Fig. 4:**
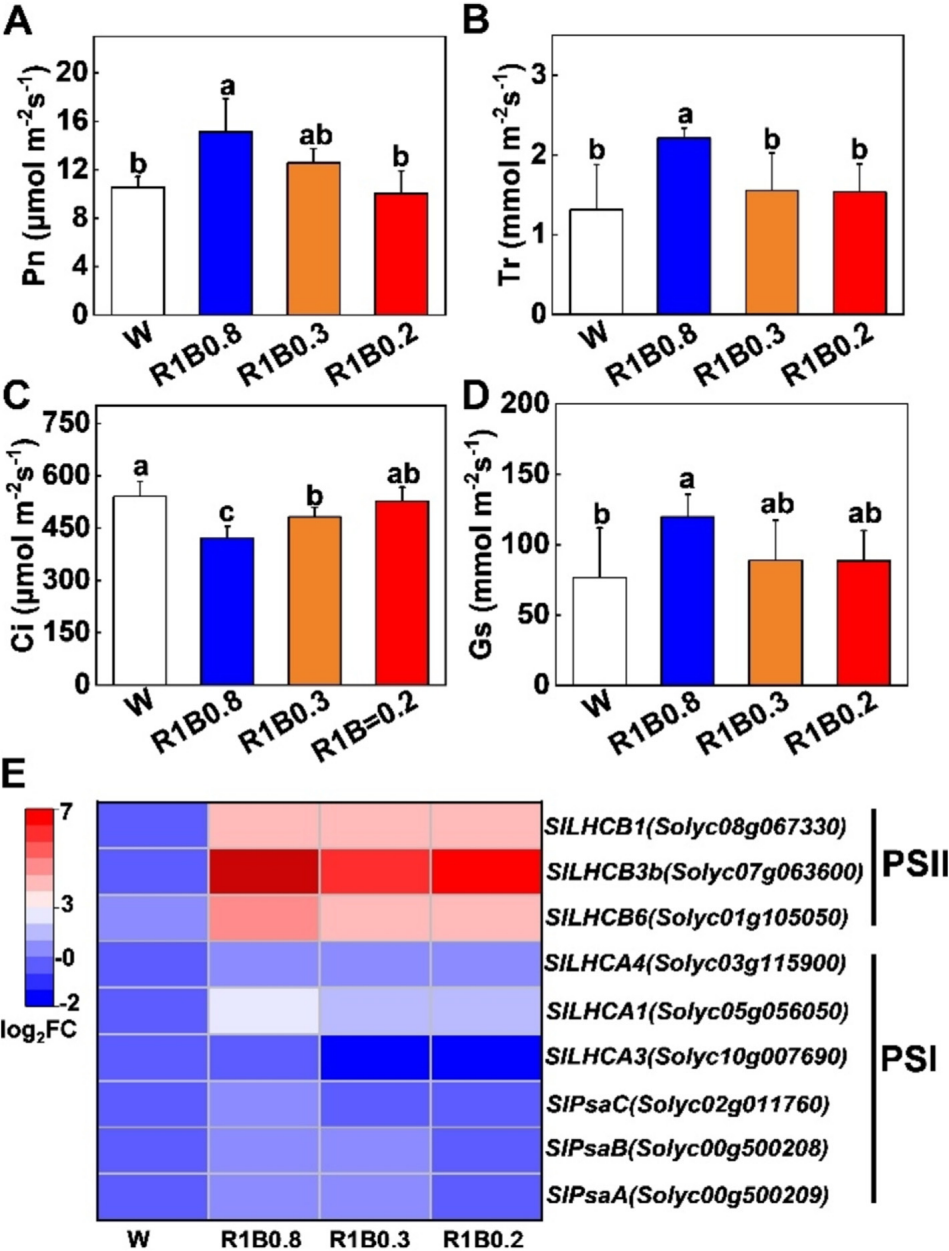
**Light quality regulates photosynthesis in tomato seedlings.** (A-D) Net photosynthetic rate, Pn (A), transpiration rate, Tr (B), internal CO_2_ concentration, Ci (C), stomatal conductance, Gs (D) in tomato leaves after exposure to W, R1B0.8, R1B0.3 and R1B0.2 light treatments for 12 d. (E) The transcript levels of light-harvesting complex genes (*LHCB/A*), and the major PSI subunit genes (*PsaC, PsaB, PsaA*) in leaves of tomato plants were determined on 2 d under W, R1B0.8, R1B0.3, R1B0.2 treatments. Values are expressed as the mean ± SD (n = 3). n = 3 independent biological replicates. At least six seedings leaves or fruits were harvested for each biological replicate. Statistical significance is annotated by different letters (*p* < 0.05).

Photosynthesis is a critical biophysical and biochemical process in nature, which has an important role in enhancing crop production. To investigate whether photosynthetic capacity was impacted by various light quality, the Pn (the net photosynthetic rate), Gs (stomatal conductance), Ci (intercellular CO2 concentration) and Tr (transpiration rate) in tomato leaves were measured under W, R1B0.8, R1B0.3, and R1B0.2 treatments. We found the Pn, Gs, and Tr were particularly increased by R1B0.8 treatments, whereas no differences in data among W, R1B0.3, and R1B0.2 lights were observed (Fig. 4A, B, D). However, the value of Ci significantly decreased under R1B0.8 treatments (Fig. 4C). Similarly, plants grown under R1B0.8 treatments showed widely induced relative transcript levels of *LIGHT- HARVESTING COMPLEX B/A* (*LHCB/A*) and the major PSI core subunit genes (*PsaC, PsaB, PsaA*) (Fig. 4E). These results show that plants exposure to R1B0.8 treatments positively regulated the expression of key photosystem core subunit genes (*SlPsaC, SlPsaB, SlPsaA*) and light-harvesting complex genes (*LHCB/A*), thereby promoting photosynthesis in tomato.

### R and B mixed lights accelerate fruit ripening and enhance fruit quality in tomato

**Fig. 5:**
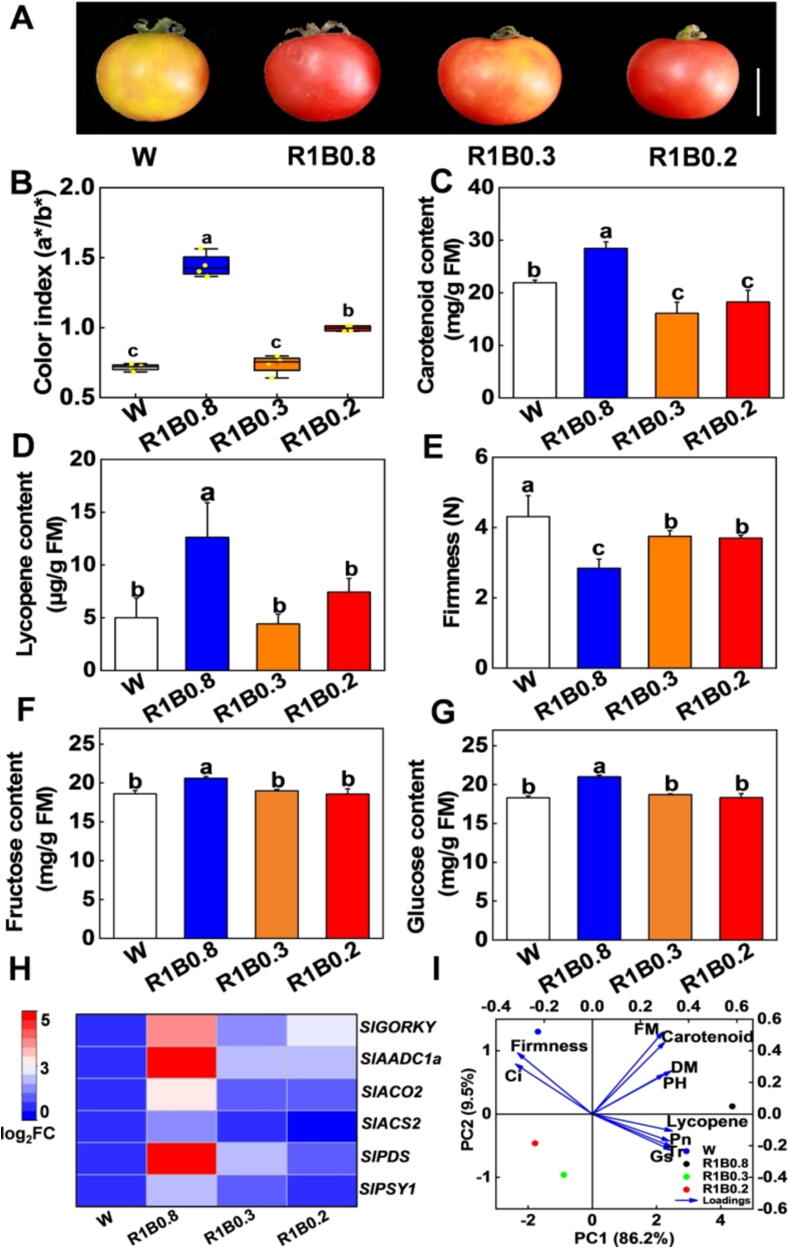
**Light quality affects fruit ripening and fruit quality in tomato.** (A) The phenotypes of tomato fruits under W, R1B0.8, R1B0.3 and R1B0.2 light treatments. Tomato fruits at the mature green (MG) stage were placed under different light quality for 10 d. Bars in (A), 2 cm. (B-G) Color index (B), carotenoid contents (C), lycopene contents (D), firmness (E), fructose contents (F), glucose contents (G) in tomato fruit after exposure to different light quality for 10 d. (H) The transcript levels of carotenoid biosynthesis genes, phytoene synthase 1 (*PSY1*)*,* phytoene desaturase (*PDS),* genes encoding ACC oxidase (*ACO2*) and ACC synthase (*ACS2*), flavor-related genes (*AADC1a)* and *GORKY* in fruits of tomato determined on 10 d under W, R1B0.8, R1B0.3, and R1B0.2 treatments. (I) Principal component analysis (PCA) of the physiological indicators for W, R1B0.8, R1B0.3 and R1B0.2 light treatment, with loadings; FM, fresh matter; DM, dry matter; PH, plant height; Pn, net photosynthetic rate; Tr, transpiration; Ci, internal CO_2_ concentration; Gs, stomatal conductance. Values are expressed as the mean ± SD (n = 3). n = 3 independent biological replicates. At least six seedings leaves or fruits were harvested for each biological replicate. Statistical significance is annotated by different letters (*p* < 0.05).

In order to explore whether the various light quality conditions impact tomato fruit ripening and fruit quality, we monitored key physiological parameters related to fruit ripening. The tomato fruits exposed to R1B0.8 treatments showed relatively red colors and higher color index (a*/b* hunter) than other light treatments such as W, R1B0.3, and R1B0.2 (Fig. 5A, B). Consistently, total carotenoid and lycopene concentrations under R1B0.8 treatments also reached the highest value compared to other light quality, with no obvious difference between W, R1B0.3 and R1B0.2 treatments (Fig. 5C, D). To further investigate the molecular regulatory mechanism of carotenoid accumulation under various light quality, the transcript levels of carotenoid biosynthesis pathway-related genes were tested by qRT-PCR. The expression of phytoene synthase 1 (*PSY1*)*,* and phytoene desaturase (*PDS*) genes were significantly upregulated under R1B0.8 treatments, which was consistent with the results that R1B0.8 promoted the carotenoid and lycopene accumulation in tomato fruits (Fig. 5H). Tomato fruits exposed to R1B0.8 light showed accelerated softening compared to W light (Fig. 5E), which was related to the up-regulation in transcript levels of ACC synthase 2 (*ACS2*) and ACC oxidase 2 (*ACO2*) (Fig. 5H). Next, we measured the soluble sugar content of tomato fruits under various light quality. Our results showed that R1B0.8 treatments significantly increased the content of fructose, glucose and soluble solids in tomato fruits (Fig. 5F, G; Supplementary Fig. S4A), whereas the same treatment did not affect the sucrose content (Supplementary Fig. S4B). Interestingly, R1B0.8 treatments induced the expression of volatile-related gene *AADC1a* (aromatic amino acid decarboxylase) and flavor-related gene *GORKY* (glycoalkaloid transporter) (Fig. 5H). Collectively, these results indicate that R1B0.8 treatments are able to promote fruit ripening and quality in tomato.

PCA analysis showed that R1B0.8 distanced away from other light treatments (W, R1B0.3, R1B0.2) in the first quadrant (Fig. 5I), indicating that R1B0.8 had an obvious difference between other treatments. The total variance explained by the 2 components was 95.7 % (PC1:86.2 %, PC2: 9.5 %). In the loading plot, we learned that samples grouped in the positive axis of PC1 according to their FM (fresh matter), carotenoid, DM (dry matter) and PH (plant height), lycopene, Pn (net photosynthetic rate transpiration), Tr (transpiration rate), Gs (stomatal conductance). The positive axis of PC2 was explained according to the firmness and Ci (internal CO_2_ concentration). Taking into consideration the percentage of variance explained in this second model, FM, DM, PH, Pn, Tr, Gs, carotenoid and lycopene were important variables. DM, Tr, Gs and lycopene contributed more to PC1 (Fig. 5I), indicating R1B0.8 promoted the growth and fruit ripening by promoting photosynthesis and lycopene.

### Photoperiod influences plant growth and photosynthesis in tomato

**Fig. 6:**
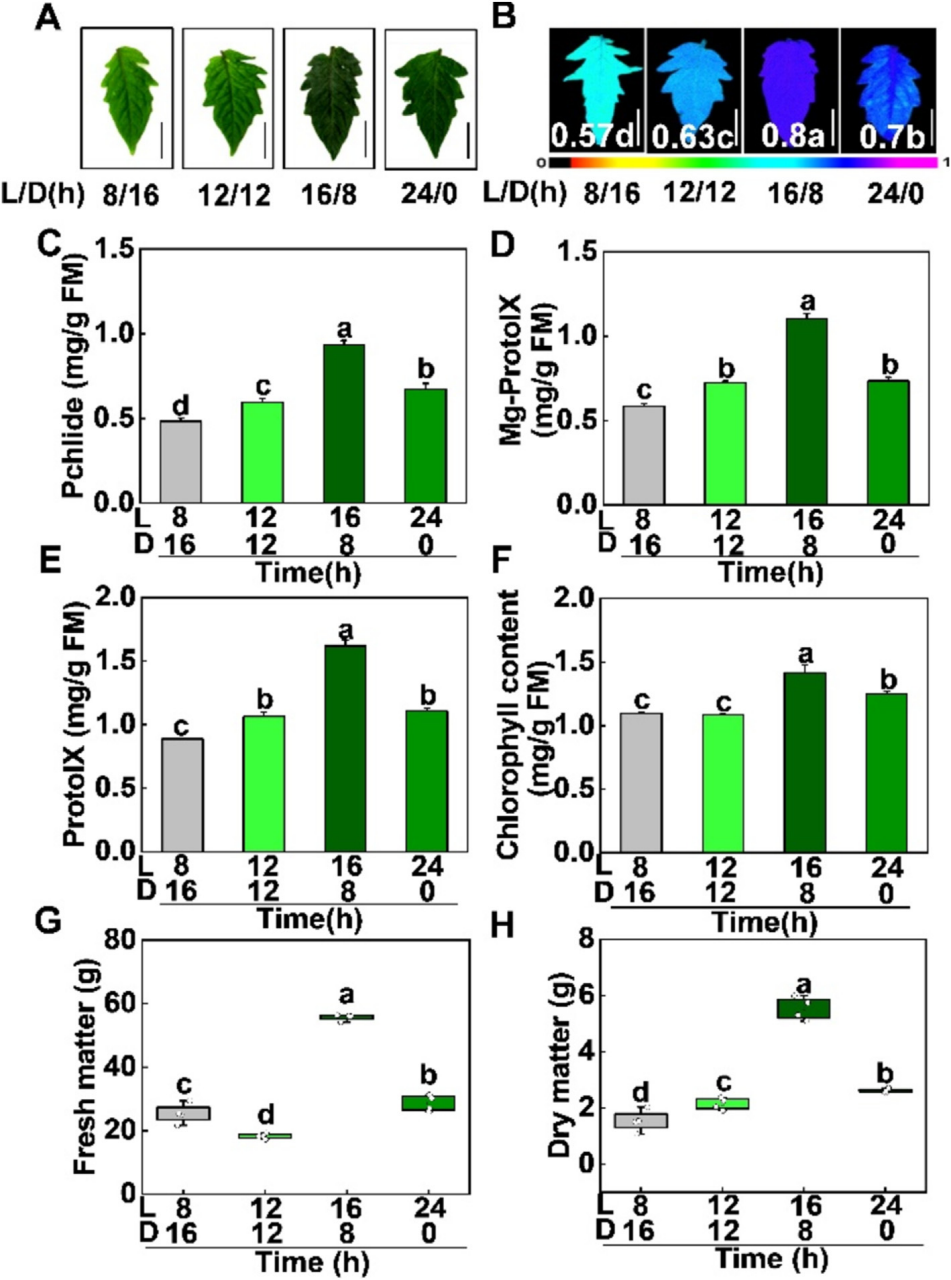
**Various photoperiods affect photosynthetic pigment contents and biomass of tomato** (A) Phenotypes of tomato leaves grown during photoperiod 8 h (8L/16D), photoperiod 12 h (12L/12D), photoperiod 16 h (16L/8D), and photoperiod 24 h (24L/0D) at R1B0.8 light treatments for 12 d. (B) The maximum quantum yield of PSII (Fv/Fm) in tomato leaves during photoperiod 8 h, 12 h, 16 h, and 24 h at R1B0.8 light treatments. Bars in (A), (B) 2 cm. (C-F) Pchlide (C), Mg-ProtoIX (D), ProtoIX (E), and chlorophyll (F) contents were measured on 12 d under different light treatments of tomato leaves. (G-H) After 12 d of light treatment fresh matter (FM; G) were measured. The dry matter (DM; H) were measured after the light treatment samples were heated at 105 °C for 30 min and then dried to constant weight at 80 °C for 4 days. Values are expressed as the mean ± SD (n = 3). n = 3 independent biological replicates. At least six seedings leaves or fruits were harvested for each biological replicate. Statistical significance is annotated by different letters (*p* < 0.05).

Although we have demonstrated that R1B0.8 light treatments is more effective to improve the yield and fruit quality, the photoperiod of R1B0.8 light treatment is not clear. Thus, four photoperiod treatments at 8/16 h, 12/12 h, 16/8h, and 24/0h (day/night) were used to further explore the optimal time of supplementary light under R1B0.8 treatments in tomato. Tomato leaves were dark green as supplementary light period increased, especially when leaves were under R1B0.8 light for 16/8h (Fig. 6A). Fv/Fm (The maximum photochemical quantum yield of photosystem II) during the photoperiod 16 h was higher than other treatments (Fig. 6B), indicating that an appropriate increase in the time of supplementary light can effectively promote the activity of photosystem II. Accordingly, tomato leaves grown under R1B0.8 light for 16/8h had the highest contents of Pchlide, Mg-ProtoIX, ProtoIX and total chlorophyll, followed by photoperiod 24/0h (Fig. 6C-F). Similarly, the value of biomass was significantly larger under R1B0.8 treatments for 16/8h than those plants under photoperiod 8/16 h, 12/12 h, and 24/0h (Fig. 6G, H; Supplementary Fig. S5). These results indicate that increasing the time of supplementary light appropriately could obviously enhance the chlorophyll content, thereby contributing to the biomass accumulation of tomato seedlings.

**Fig. 7:**
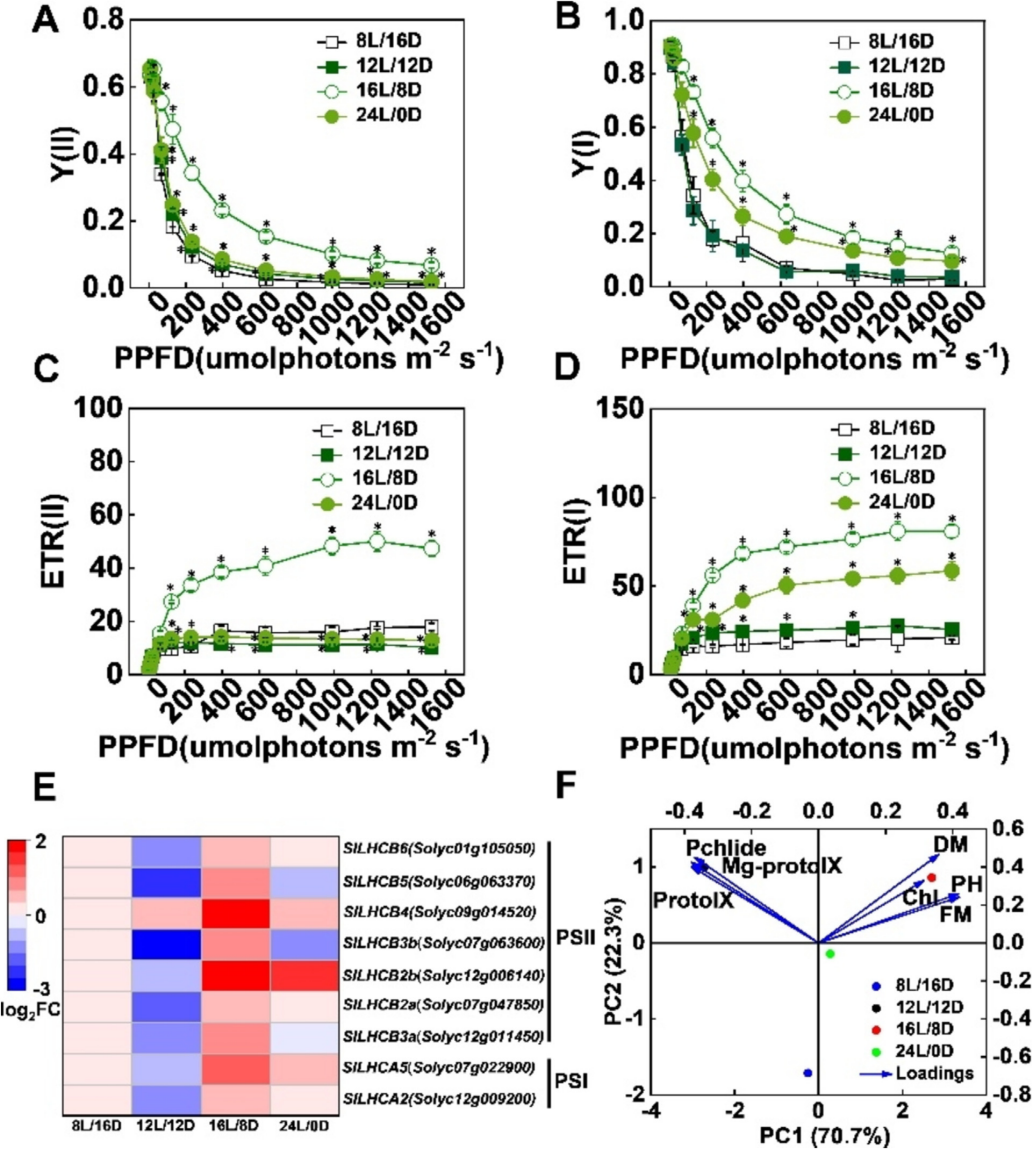
**Photoperiod influences the photosystem efficiency in tomato seedlings**. (A-D) [Y(II), A], [Y(I), B], [ETR(II), C] and [ETR(I), D] after tomato exposure to the photoperiod 8 h (8L/16D), photoperiod 12 h (12L/12D), photoperiod 16 h (16L/8D), photoperiod 24 h (24L/0D) at R1B0.8 light treatments for 12 d. (E) The transcript levels of *LHCB/A* were measured on 2 d under various photoperiods of tomato plants. (F) Principal component analysis (PCA) of the physiological indicators for photoperiod 8 h (8L/16D), photoperiod 12 h (12L/12D), photoperiod 16 h (16L/8D), photoperiod 24 h (24L/0D) at R1B0.8 light, with loadings; FM, fresh matter; DM, dry matter; PH, plant height; Chl, chlorophyll. Values are expressed as the mean ± SD (n = 3). n = 3 independent biological replicates. At least six seedings leaves or fruits were harvested for each biological replicate. Asterisks indicate significant difference (*p* < 0.05).

To further explore the relationship between photoperiod and photosynthetic electron transport, we next measured the photochemical parameters under the photoperiod 8/16 h, 12/12 h, 16/8h and 24/0h in tomato seedlings. Plants exposed to the photoperiod 16/8h exhibited the highest value of Y(I), Y(II), ETR(I) and ETR(II) (Fig. 7A-D). In addition, the expression levels of *LHCAs* and *LHCBs* genes were remarkably induced by the photoperiod 16/8h (Fig. 7E), suggesting that an appropriate increase in the time of supplementary lighting could notably improve the photosynthetic capacity in PSI and PSII by upregulating the transcript levels of *LHCAs* and *LHCBs*.

PCA analysis showed that R1B0.8 for 16/8h distanced away from other treatments (8/16h, 12/12 h, 24/0h) in first quadrant (Fig. 7F), indicating that R1B0.8 for 16/8h have an obvious difference between other treatments. In loading plot, we learned that and PH (plant height), FM (fresh matter) are important variables (Fig. 7F), suggesting that PH and FM were important for R1B0.8 under 16/8h promoting the growth and development in tomato.

## Discussion

Tomato yield and quality are sensible to modulation of external factors, such as light, water, exogenous substances, etc [3], [19], [33]. The application of exogenous substances, such as biostimulants and fertilizers, could promote fruit ripening, size, and nutraceutical values [41], [42]. In addition, a previous study also showed that plants grown in an aquaponics system had a higher total phenolic content and antioxidant capacity than organic soil-grown crops [43]. As known, light is an indispensable energy source that drives efficient photosynthesis to achieve optimal yield of crops. Plants frequently monitor changes in the dynamic fluctuations in light environments and fine-tune their growth patterns and development correspondingly to optimize fitness. However, plants respond differently to spectral wavelengths of light, and each plant species has a specific optimal R and B light ratio for metabolism and physiological development [44]. Here, we revealed that R1B0.8 treatments significantly stimulated the biomass in tomato seedlings (Fig. 1G, H; Supplementary Fig. S1). These results corresponded to most of the studies that increasing the proportion of B light could promote stomatal opening, thereby increasing photosynthesis and biomass accumulation [11]. Moreover, compared with R1B0.8 treatments, W light reduced FM and DM in tomato plants mainly because W light also contains yellow (Y, 580–595 nm) and green light (G, 500–560 nm). However, Y and G lights have less effect on plant growth and development compared to R and B monochromatic lights [45]. Hence, manipulating the R and B light ratio is vital to enhance the fresh and dry matter accumulation in tomato plants.

Chlorophyll is the major photosynthetic pigment used for capturing light energy, which is the material basis for photosynthesis in plants [46]. Prior studies have shown that increasing the ratio of B light is beneficial to chlorophyll biosynthesis in horticultural crops [9]. In our study, compared with W light, chlorophyll contents significantly increased in tomato leaves under R1B0.8 treatments, while no obvious difference in carotenoid accumulation was found (Fig. 1C-E). Consistently, we also found that R1B0.8 light was more conducive to enhancing Gs, resulting in a higher rate of Pn than W, R1B0.3 and R1B0.2 treatments (Fig. 4A, D). These results indicated that appropriately increasing B light ratios under R light conditions could enhance chlorophyll accumulation, which absorbed more light energy to improve photosynthetic rates [47]. Furthermore, the photosynthetic rates increased with the increase in B light ratio (Fig. 4), which might be due to the Rubisco, Cyb_6_f complex and PSII protein accumulation [48]. In addition, R1B0.8 treatments enhanced photosynthesis was also related to the relatively high expression levels of *LHCB*, *LHCA* and PSI subunit genes (*SlPsaC, SlPsaB, SlPsaA*) (Fig. 4E). The *LHC* family genes controls the assembly of PSI and II light-harvesting complex, which play a critical role in photosynthesis, plant growth and development [49]. Previous studies showed that B light photoreceptors (phototropins and cryptochrome) directly perceive B light, thereby activating a signaling cascade that leads to the rapid opening of stomata and thus promotes the photosynthesis of plants [50].

Destruction of any component of the photosynthetic machinery, including photosynthetic pigment, PSI and PSII, the electron transfer chain, and the CO_2_ reduction pathway, is enough to hinder the entire photosynthesis process in plants. PSII, in particular, is the most sensitive to environmental change and most vulnerable to photodamage [51]. It is well known that the imbalance of available photons in PSI and PSII when plants are exposed to long-term monochromatic R light, and long-term lack of B light reduced photosynthetic performance [16]. Here, we found that plants grown under R1B0.8 light could induce an increase in Y(II) in tomato leaves (Fig. 2A), thereby enhancing the efficiency of PSII energy capture and increasing the activity of PSII reaction centers, implying that plants grown under higher ratio of B light are less susceptible to light damage [16]. The highest qP and ETR(II) were found in tomato plants under R1B0.8 light, suggesting that B light enhanced PSII function and increased energy of the excitation reaction center, which was beneficial to improve the photosynthetic efficiency [52] (Fig. 2B, C). Meanwhile, a typical OJIP phase was shown in tomato plants grown under all light treatments (Fig. 3A), indicating that photosynthesis is normal in the plants. R1B0.8 treatments increased the fluorescence intensity of the J-I-P phase, which indicated that R1B0.8 treatments could promote the electron transfer from Q_A_ to Q_B_ of PSII [53]. The performance index of PSII (PI_ABS_) is reported to serve as a more sensitive indicator of overall PSII-related energy fluxes and photosynthesis [54]. The increase in PI_ABS_ by the promotion of electron transfer is a result of the increased function of PSII [55]. In our study, the highest PI_ABS_ value was observed under R1B0.8 treatments (Fig. 3B), which is in line with other photosynthetic paraments, implying that higher photosynthesis efficiency and better vigor of plants under the optimal rate of R and B light. In addition, the improvement of photosystem performance by R1B0.8 light is mainly due to an increase in the number of reaction centers, and energy dissipation, as evidenced by RCs/CSm, DI0/CSm (Fig. 3C). Similarly, the higher inhibitory effect on the re-oxidation of QA- to QA leads to an increase in ET0/CSm under R1B0.8 light [56] (Fig. 3C). Taken together, manipulation of light quality is of great significance for promoting the PSII photochemical efficiency and improving the light energy utilization rate, so as to increase production in tomato.

The ripening of tomato fruit is a complex genetic process that occurs through a series of physiological and biochemical changes, including the alterations in color, texture and flavor of the fruits [57]. Transcriptomics and metabolomics analyses also have shown that light signaling regulation of tomato fruit ripening is mainly mediated through carotenoid accumulation, ethylene biosynthesis and signal transduction pathways [58], [59], [85]. In addition, previous studies also demonstrated that B light receptor CRY1a could increase the accumulation of phytoene, lycopene, and β-carotene in tomato fruits, implying that *CRY1a*-mediated B light signaling is essential for regulating the color of fruits [60]. In addition, B light also induced the lycopene metabolism process of fruits via up-regulating the expression of light-regulated transcription factors such as *SlPIFs* and *SlHY5* [61]. Here, we observed that R1B0.8 treatments promoted lycopene and carotene accumulation (Fig. 5A-D), and upregulated carotene biosynthesis-related genes, such as *SlPSY1* and *SlPDS* in tomato fruits (Fig. 5H). In agreement with this, R and B mixed lights sharply improved lycopene biosynthesis and carbohydrates in tomato fruits [25]. In particular, it has been suggested that ethylene biosynthesis and the change in transcription of ethylene biosynthesis genes were significantly regulated by light quality [62], [63]. Our results showed that the fruits under R1B0.8 treatments could largely upregulate the expression of *SlACO2* and *SlACS2* (Fig. 5H, D), which in turn significantly reduces the firmness in tomato fruits (Fig. 5E). In agreement with this, B light strongly induced the expression of *PpACO1*, and *PpACS3*, which play a positive role in ethylene biosynthesis, thus promoting the softening of fruits in peaches [64]. Fruit firmness is closely linked to fruit ripening, and previous studies had shown that ethylene treatment could accelerate fruit softening, but 1-methylcyclopropene (1-MCP), a competitive ethylene signaling inhibitor, had an opposite effect on fruit softening [65]. The *SlACS2* silenced fruit showed significantly inhibited ethylene emission and delayed the fruit ripening in tomato [66]. Light signaling transcription factors SlHY5 can directly bind to the promoter of ethylene biosynthesis genes to promote fruit ripening [67]. CUL4 (CULLN4) and COP1 (CONSTITUTIVELY) were negative regulators in tomato fruit ripening [68], [69]. Interestingly, the light-hyperresponsive *hp2* (*high pigment)* mutant ripening fruits can reduce the ethylene emission and increase the expression of genes encoding master regulators of ripening [70]. In addition, the light regulatory network on fruit ripening is complex, and other factors related to the process, such as epigenetic mechanisms, abscisic acid and auxin signaling, also play a vital role in light regulation of fruit ripening [71], [72], [73]. Our results suggested that light quality influences fruit ripening via the accumulation of carotene and ethylene emissions. Future work will further focus on the relationship between the R and B mixed light and the function of other factors, like epigenetic mechanisms and auxin signaling, in fruit ripening.

Furthermore, light quality has generally been reported to participate in regulating the soluble sugar accumulation in plants. For instance, FR radiation increases the sinking strength of the fruit primarily by simultaneous upregulation of sugar transport and metabolism, thereby stimulating the distribution of dry mass to the fruit [74]. B light-enhanced soluble sugar accumulation might be related to photosynthetic carbon partitioning, as well as to the number of stomata, showing a higher sensitivity to B light [75]. Previous studies displayed the lowest content of soluble sugars under long-term monochromatic B light in tomato plants [76], and properly supplemented R significantly increased the contents of fructose and glucose [77]. Here, we clarified that R and B mixed light (R1B0.8) might induce a higher content of soluble sugar, especially fructose and glucose (Fig. 5F, G), which may be attributed to the enhanced photosynthesis by B light, resulting in more sugar input into the fruits. In addition, Light quality had a significant differential effect on the transformation and formation pathways of volatile metabolites [78]. In our study, the expression of flavor-related gene *SlGORKY*, and volatile-related gene *SlAADC1a* were upregulated under R1B0.8 light treatments (Fig. 5H), suggesting that light regulation of these genes can be an important way for improving the fruit quality of tomato. Overall, R and B mixed light can regulate fruit ripening and fruit quality in tomato. However, how light quality regulates the specific mechanisms of fruit development and ripening, especially R and B mixed light remains an interesting topic for future investigation. In addition, light also has a significant effect on nutritional attributes during fruit ripening from transcriptomics and metabolomics analyses [59]. PHY-dependent light perception positively regulates tocopherol production during tomato fruit ripening [29]. B light also promotes ascorbate (vitamin C) synthesis by inactivating the PAS/LOV photoreceptor that inhibits GDP-L-galactose phosphorylase [13]. Thus, it is necessary to systematically explore how light quality affects other nutritional attributes like vitamin and taste in the future.

The changes in light conditions (including photoperiod and light quality) have a profound influence on the morphogenesis and physiological metabolism in plants [26]. Manipulation of light quality and photoperiod is currently applied as a common agronomic technique to improve horticultural crop production. Therefore, we subsequently investigated how the photoperiod regulates the growth and development of tomato plants. The process of chlorophyll synthesis starts with the formation of glutamyl-tRNA, to chlorophyll *a* and *b* is synthesized through several precursors. PrototypeIX, Mg-ProtoIX and Pchlide are key precursors in the chlorophyll synthesis process [79]. Here, we found that plants grown under R1B0.8 light for 16h accumulated increased levels of total chlorophyll, Pchlide, Mg-ProtoIX, and ProtoIX (Fig. 6A, C-F), suggesting that photoperiod 16 h could efficiently aid tomato leaves to convert, capture and transfer light energy [80]. However, plants grown under photoperiod 24 h also displayed a relatively lower chlorophyll content than 16h (Fig. 6A, F). It may be because of the disruption of plant circadian rhythm, which causes photoinhibition. Exposure to a photoperiod of 16 h may improve the efficiency of PSI and PSII by increasing the electron transport rates and photosystem energy capture (Fig. 7A-D), resulting in the promotion of the biomass of tomato plants (Fig. 6G, H; Supplementary Fig. S5). Moreover, the expression of *LHCB/A* was significantly induced by photoperiod 16 h (Fig. 7E). Thus, an appropriate increase in the time of supplementary light in the photoperiod positively affects the electron transfer efficiency, photosynthetic capacity and biomass in plants [81], [82], [83].

## Conclusions

**Fig. 8:**
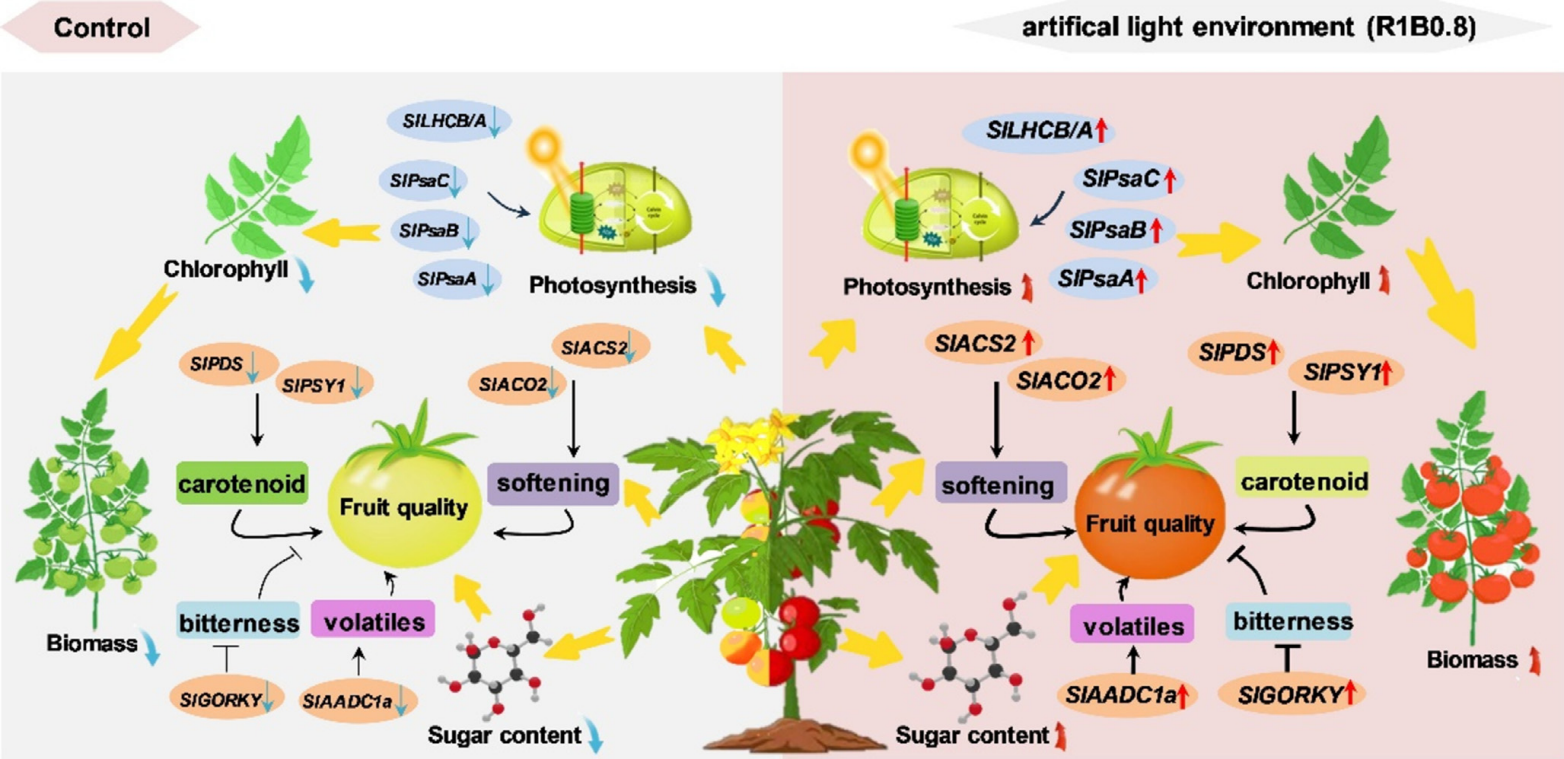
**Schematic representation outlining the role of the combination of red and blue light in regulation of biomass and fruit nutrition in tomato.** Manipulation of artificial light environment and photoperiod can increase plant biomass through improving the accumulation of chlorophyll, promoting photosynthesis, the activity of photosystem and inducing the transcript levels of major PSI subunit genes (*SlPsaC, SlPsaB, SlPsaA*) and light-harvesting complex genes (*SlLHCB/A*). In addition, R1B0.8 light accelerates the fruit ripening process and promotes fruit quality via increasing the transcript levels of carotenoid biosynthesis genes (*SlPSY1, SlPDS*), ethylene biosynthesis genes (*SlACO2, SlACS2*), volatile-related gene (*SlAADC1a*), and flavor-related gene (*SlGORKY*). In brief, supplementation with R1B0.8 for 16 h could prominently improve photosynthetic pigment accumulation, photosystem efficiency, and biomass in tomato.

Our study demonstrates the important role of the combination of red and blue light on the photosynthetic performance and development of tomato. Red and blue mixed light (R1B0.8) under 16/8h (light/dark) photoperiod increased the accumulation of chlorophyll, promoted photosynthesis and photosystem efficiency by improving the light energy utilization rate and upregulating the expression of photosystem core subunit genes (*SlPsaC, SlPsaB, SlPsaA*) and light-harvesting genes (*SlLHCB/A*), resulting in increased plant biomass accumulation. Meanwhile, R1B0.8 light also induced carotenoid accumulation and accelerated fruit ripening, which was associated with the upregulation of carotenoid biosynthesis genes (*SlPSY1, SlPDS*) and ethylene biosynthesis genes (*SlACS2, SlACO2*) in tomato. Consistently, fruits exposed to R1B0.8 treatments contained increased levels of fructose and glucose, the major index of fruit quality, with significantly upregulated expression of *SlAADC1a* and *SlGORKY* genes (Fig. 8). The study screened out the optimal light environment that could promote plant growth and fruit quality, thereby providing a theoretical basis for manipulating the light environment in protected horticultural production facility.

## CRediT authorship contribution statement

**Ying Zhang:** Investigation, Validation, Data curation, Formal analysis, Writing – original draft. **Kangyou Zhu:** Investigation, Validation, Data curation, Formal analysis, Writing – original draft. **Xiujie Wang:** Validation, Data curation, Formal analysis. **Jiarong Yan:** Validation, Data curation, Formal analysis. **Haiyan Zhu:** Methodology. **Nan Zhang:** Methodology. **Yiting Wang:** Methodology. **Qi Zhao:** Methodology. **Yanan Liu:** Methodology. **Xin Bu:** Methodology. **Chenghao Jiang:** Validation, Data curation. **Xin Sun:** Formal analysis, Writing – review & editing. **Golam Jalal Ahammed:** Writing – review & editing. **Shuyu Cai:** Formal analysis. **Sida Meng:** Formal analysis. **Zhouping Sun:** Formal analysis, Funding acquisition. **Mingfang Qi:** Formal analysis. **Tianlai Li:** Conceptualization, Supervision, Writing – review & editing. **Feng Wang:** Conceptualization, Supervision, Funding acquisition, Project administration, Writing – review & editing.

## Declaration of competing interest

The authors declare that they have no known competing financial interests or personal relationships that could have appeared to influence the work reported in this paper.
