# Supplementary material for: Manipulation of artificial light environment improves plant biomass and fruit nutritional quality in tomato
Source: J Adv Res. 2024 Nov 24;75:79–93. doi: 10.1016/j.jare.2024.11.030 (PMC12536594; doi:10.1016/j.jare.2024.11.030)

Supplementary Table S1. List of primer sequences used for qRT-PCR analysis.

| **Gene** | **Accession number** | **Forward primer (5’-3’)** | **Reverse primer (5’-3’)** | |
| --- | --- | --- | --- | --- |
| ***SlPsaA*** | Solyc00g500209 | GCACTAGGCCCAATGTGAGT | | TTTCGAGGAATGGGCCAGAC |
| ***SlPsaB*** | Solyc00g500208 | AGAGGGGAGTACGTTCGGTG | | AGAGGGGAGTACGTTCGGTG |
| ***SlPsaC*** | Solyc02g011760 | ACCTTGGGACGGTTGTAAGG | | TCTGTTGGACAGGCGGATT |
| ***SlLHCB*** | Solyc08g067330 | TGACTGGTGACTACGGATGG | | TAGGCTAATAGCTCGGGAAA |
|  | Solyc07g063600 | GCTCAGCCACAGTTGTTAGA | | CAGGTCCATACCACAAATCA |
|  | Solyc01g105050 | ATGACCACCACATCTGCTACT | | GCTGCCACAACAAACCTCTT |
|  | Solyc06g063370 | CCCAGAAGCCTTCAACAAAT | | CACCACCAACAAGAACAACC |
|  | Solyc09g014520 | CAACTCCGTTTCAGCCTTAC | | CAACATAGCCCATCTTCCAT |
|  | Solyc12g006140 | GGAGAAGATAGGCCAAAGTA | | CAGTGTCCCATCCATAGTCA |
|  | Solyc07g047850 | TTTTCGGAGCAAACACCATC | | GCACCAAGCATAGCCCAACG |
|  | Solyc12g011450 | TGGGACACTGCTGGTTTATC | | CACCGTCACTGAAGATTTGT |
|  | Solyc03g115900 | TGATTATCTTGATGGCAGTCT | | AACGACCATTCACCAGTTCAGC |
| ***SlLHCA*** | Solyc05g056050 | CACCGGAGTTACCAACGACA | | AACAGCAAGCATAGCCCATC |
|  | Solyc10g007690 | CCCACCAGCAGGAACATACA | | ACCTGACCCACCCAAACCCT |
|  | Solyc07g022900 | ACTAGAAGCTGCACTGGAAG | | GAGTAACAGAAGCCTGAACG |
|  | Solyc12g009200 | AATCAGGGAGCTACCTACAA | | CTTCTTTACCAGCATACAATCT |
| ***SlPSY1*** | Solyc03g031860 | CGATGGTGCTTTGTCCGATAC | | CTCATCAACCCAACCGTACC |
| ***SlPDS*** | Solyc03g123760 | CGTTCCGTGCTTCTCCGC | | CTAGAACATCCCTTGCCTCCAG |
| ***SlACO2*** | Solyc12g005940 | TTTATTACAAAGTGTGCGTCCCTA | | CTCATTTTTGGGTATTAAAATATGT |
| ***SlACS2*** | Solyc01g095080 | GGCTACTAATGAAGAGCATGGC | | GACCCATTTGGATAACTCCGTTG |
| ***SlAADC1a*** | Solyc08g068680 | ACCATGAATTTGTGCGTCGT | | TCTCGTGTGATTCCTGGCAT |
| ***SlGORKY*** | Solyc03g120570 | GGAACCTTTGCACAGTGGAG | | ATCATGATCCCCGTTGACCA |
| ***ACTIN2*** | Solyc11g005330 | TGTCCCTATTTACGAGGGTTATGC | | CAGTTAAATCACGACCAGCAAGAT |

Supplementary Table S2. Definition of terms and formulae for calculation of the JIP-test parameters from the Chl a fluorescence transient OJIP emitted by dark-adapted leaves.

| **Fluorescence parameters** | **Description** |
| --- | --- |
| ψ_0_=T_0_/TR_0_ =(1–V_J_) | probability (at t = 0) that a trapped exciton moves an electron into the electron transport chain beyond Q_A_− |
| 1-V_I_=1-(F_I_–F_0_)/(F_M_–F_0_) | reflects the content of PSI reaction centers as well as the electron flow capacity from plastoquinone to the PSI electron acceptors |
| OEC=[1-(V_K_/V_J_)_treat_]/[1-(V_K_/V_J_)_control_]×100 | the fraction of O_2_ evolving centre (OEC) |
| ϕ_Eo_=ET_0_/ABS=[1–(F_0_/F_M_)]ψ_0_ | quantum yield of electron transport (at t = 0) |
| PI_ABS_=(RC/ABS)[ϕ_Po_/(1–ϕ_Po_)][ψ_0_/(1–ψ_0_)] | performance index (potential) for energy conservation from photons absorbed by PSII to the reduction of intersystem electron acceptors |
| PI_total_=(PI_ABS_)×[δ_Ro_/(1–δ_Ro_)] | performance index (potential) for energy conservation from photons absorbed by PSII to the reduction of PSI end acceptors |
| ϕ_Ro_=(1–F_0_/F_M_)(1–V_I_) | quantum yield for reduction of end electron acceptors at the PSI acceptor side (RE) |
| δ_Ro_=(1–V_I_)/(1–V_J_) | efficiency/probability with which an electron from the intersystem electron carriers moves to reduce end electron acceptors at the PSI acceptor side (RE) |
| V_K_/V_J_=[(F_300_–F_0_)/(F_M_–F_0_)]/[(F_J_–F_0_)/(F_M_–F_0_)] | a relative measure of inactivation of OEC and/or the functional antenna size |
| V_K_=(F_300_–F_0_)/(F_M_–F_0_) | relative variable fluorescence at time K-step (F_300μs_, fluorescence at 300 μs) |
| V_J_=(F_J_–F_0_)/(F_M_–F_0_) | relative variable fluorescence at time J-step (FJ ≡ F_2ms_, fluorescence at the 2 ms of O-J-I-P) |


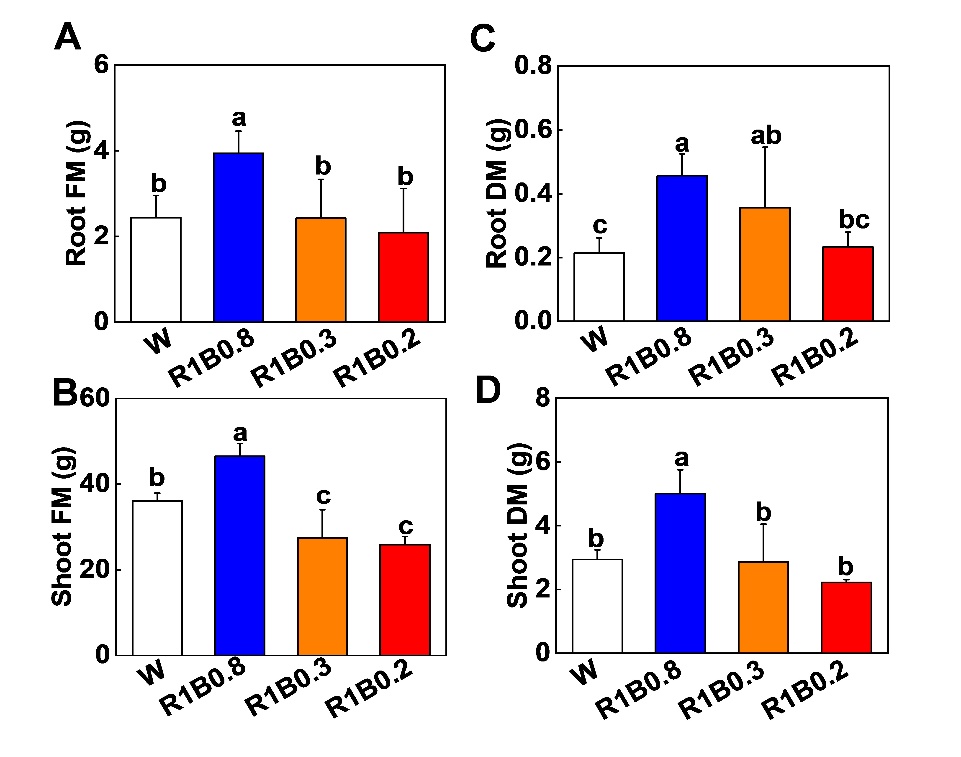


**Supplementary** Fig. S1**.** **Various light environments influence biomass accumulation in tomato seedlings.** (A-D) After 12 d of light treatment root fresh matter (A), shoot fresh matter (B) were measured. The root dry matter (C) and shoot dry matter (D) were measured after the light treatment samples were heated at 105 ^o^C for 30 min and then dried to constant weight at 80 ^o^C for 4 days. Values are expressed as the mean ± SD (n=3). n=3 independent biological replicates. At least six seedings leaves or fruits were harvested for each biological replicate. Statistical significance is annotated by different letters (*p*<0.05).

**Supplementary** Fig. S2**.** **Various light environments** **affected photosynthetic pigment contents and biomass in LYDL tomato plants**. (A-D) chlorophyll *a* (Chl *a*; A), chlorophyll *b* (Chl *b*; B), total chlorophyll (Total Chl; C), and carotenoid contents (D) in tomato leaves after tomato plants exposure to W, R1B0.8, R1B0.3 and R1B0.2 light treatments for 12 d. (E-F) After 12 d of light treatment fresh matter (FM; E) were measured. The dry matter (DM; F) were measured after the light treatment samples were heated at 105 ^o^C for 30 min and then dried to constant weight at 80 ^o^C for 4 days. Values are expressed as the mean ± SD (n=3). n=3 independent biological replicates. At least six seedings leaves or fruits were harvested for each biological replicate. Statistical significance is annotated by different letters (*p*<0.05).


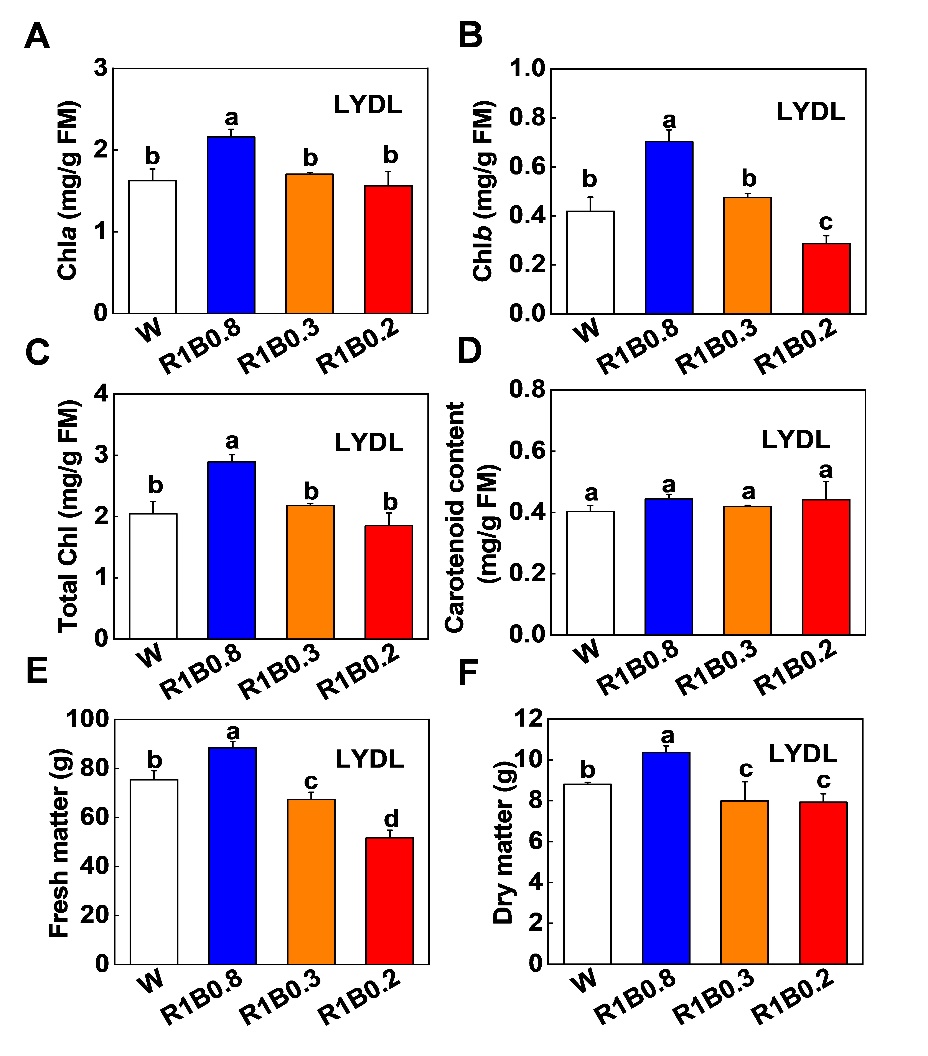


**Supplementary** **Fig. S3. Light quality affect chlorophyll precursor contents in tomato.** (A-C) Pchlide (A), Mg-ProtoIX (B), ProtoIX (C) contents were measured during photoperiod 8 h (8L/16D), photoperiod 12 h (12L/12D), photoperiod 16 h (16L/8D), and photoperiod 24 h (24L/0D) at R1B0.8 light treatments for 12 d. Values are expressed as the mean ± SD (n=3). n=3 independent biological replicates. At least six seedings leaves or fruits were harvested for each biological replicate. Statistical significance is annotated by different letters (*p*<0.05).


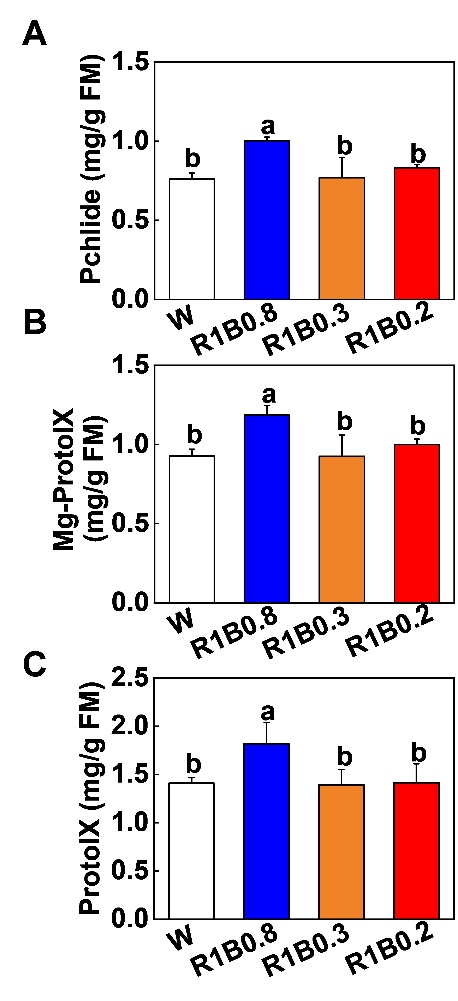


**Supplementary** **Fig. S4. R and B mixed light affects fruit quality in tomato.** (A-B) soluble solids (A), sucrose (B) contents in tomato fruit after exposure to different light quality for 10 d. Values are expressed as the mean ± SD (n=3). n=3 independent biological replicates. At least six seedings leaves or fruits were harvested for each biological replicate. Statistical significance is annotated by different letters (*p*<0.05).


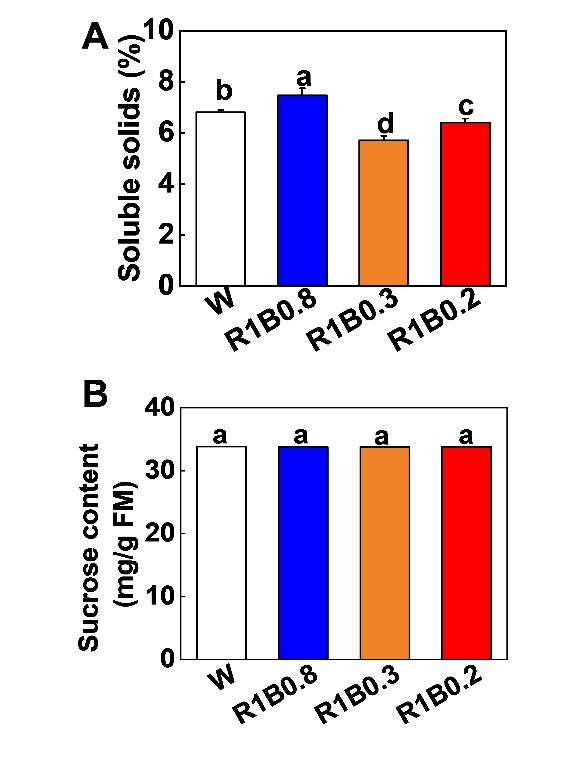


**Supplementary** **Fig. S5. Various photoperiods influence biomass and growth in tomato seedlings**. (A-D) After 12 d of light photoperiod root fresh matter (A), shoot fresh matter (C) were measured. The root dry matter (B) and shoot dry matter (D) were measured after the light treatment samples were heated at 105 ^o^C for 30 min and then dried to constant weight at 80 ^o^C for 4 days. (E-F) Plant height (E) and Stem diameter (F) in tomato seedlings at the five-leaf stage after transferring to different photoperiod for 12 d. Values are expressed as the mean ± SD (n=3). n=3 independent biological replicates. At least six seedings leaves or fruits were harvested for each biological replicate. Statistical significance is annotated by different letters (*p*<0.05).


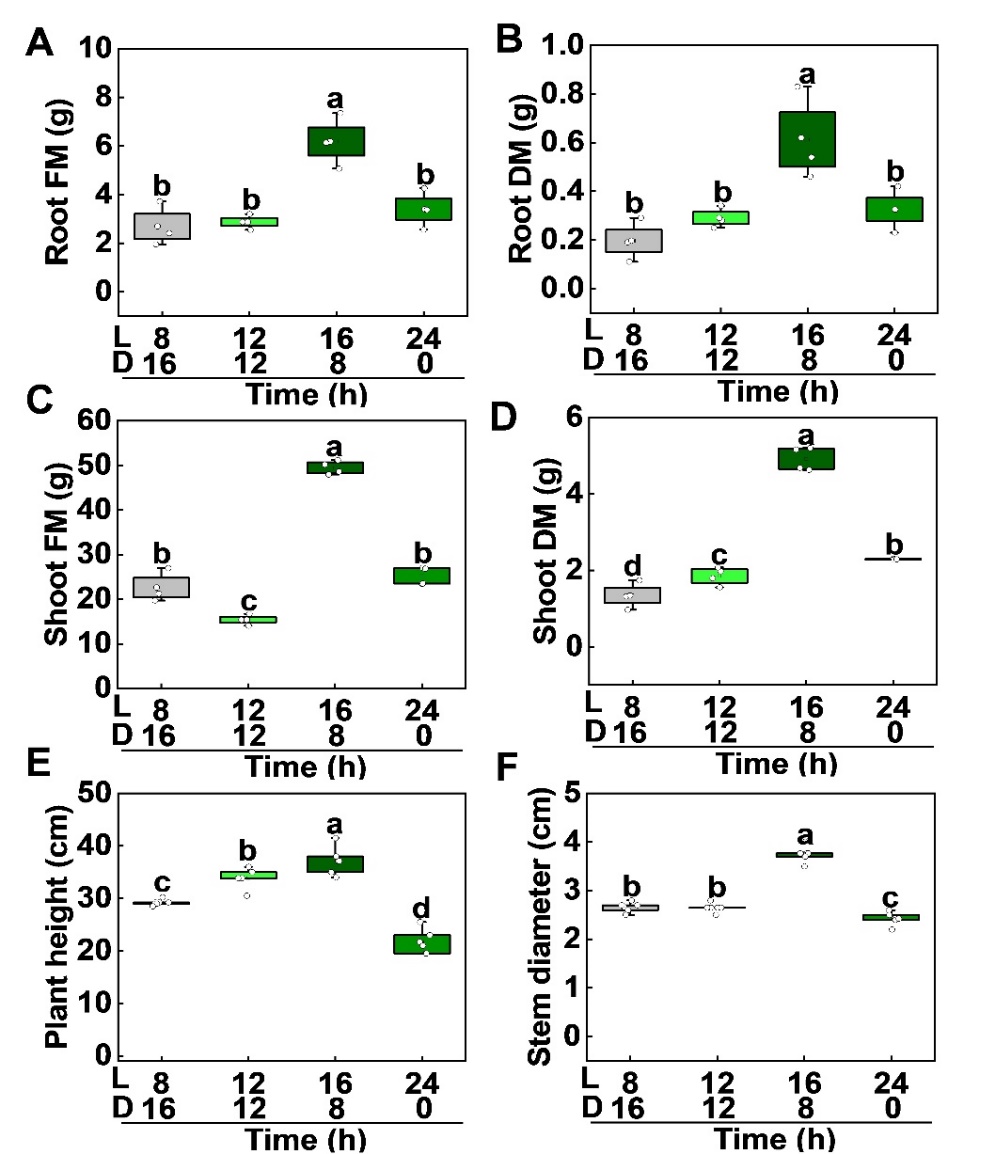

Supplement: Supplementary Data 1 [file mmc1.docx]
